# A non-synonymous polymorphism in IL-23R Gene (rs1884444) is associated with reduced risk to schistosomiasis-associated Immune Reconstitution Inflammatory Syndrome in a Kenyan population

**DOI:** 10.1186/1471-2334-14-316

**Published:** 2014-06-10

**Authors:** George O Ogola, Collins Ouma, Walter GZO Jura, Erick O Muok, Robert Colebunders, Pauline N Mwinzi

**Affiliations:** 1 Centre for Global Health Research, Kenya Medical Research Institute, Kisumu, Kenya; 2 Maseno University, Maseno, Kenya; 3 Department of Clinical Sciences, Institute of Tropical Medicine, Antwerp, Belgium

**Keywords:** HAART, IRIS, Non-synonymous, SNP, Schistosomiasis

## Abstract

**Background:**

Human Immunodeficiency Virus (HIV) and Schistosomiasis co-infection is common among residents at the shores of Lake Victoria in Kenya. About 36% of this population initiating antiretroviral therapy (ART) experience Immune Reconstitution Inflammatory Syndrome (IRIS) that complicates recovery. Several IL-23R alleles have been associated with susceptibility to both autoimmune and inflammatory diseases through T-helper type 17 (TH_17_) cells. However, whether or not variants within the IL-23R increase susceptibility to IRIS in western Kenya is unknown. The objective of the current study was to determine the association between IL-23R gene polymorphisms, CD4+ cell counts and HIV RNA levels and IRIS in HIV and *Schistosoma mansoni* co-infected patients undergoing highly active anti-retroviral therapy (HAART).

**Methods:**

A three-month case–control study was conducted on antiretroviral naïve schistosomiasis/HIV co-infected fishermen starting HAART in Uyoma Rarieda, Siaya County, Kenya. Seventy one patients were sampled at baseline and followed up for three months, to establish if they developed *Schistosoma*-related IRIS. In addition, the CD4+ cell counts and HIV RNA levels were determined in pre- and post-administration of HAART. Variations at five polymorphic sites of IL-23R (rs1884444, rs11465754, rs6682925, rs7530511 and rs7539625) based on >10% minor allele frequency in Yoruban reference population was determined using Allelic Discrimination Assay. The association between the five variants and susceptibility to IRIS was determined using logistic regression while controlling for potential confounders. In addition, the functional differences between the baseline CD4 + Cell counts and viral loads were determined using medians while across IL-23R genotypes were determined using Kruskal-Wallis tests.

**Results:**

Overall, 26 (36.6%) patients developed schistosomiasis-associated IRIS at a median age of 35.5 years. Carriage of the TT genotype at the non-synonymous rs1884444 T > G relative to GG, was associated with a decreased risk of schistosomiasis-associated IRIS (OR, 0.25, 95% CI, 0.07-0.96, *P* = 0.043) while both baseline CD4+ cell counts and viral loads had no association with IRIS.

**Conclusion:**

These findings indicate that the non-synonymous variant rs1884444 T > G of IL-23R is associated with a decreased risk to schistosomiasis-associated IRIS. However, additional studies in a larger cohort and with an all inclusive polymorphic variants in the synonymous and non-synonymous regions need to be evaluated.

## Background

Despite the declining incidence of AIDS-related mortality over past decades, it still remains one of the most common causes of death, particularly in sub-Saharan Africa, where resources are limited [1]. A significant number of AIDS cases develop into severe clinical manifestations described as Immune Reconstitution Inflammatory Syndrome (IRIS) during the first few months of starting highly active antiretroviral therapy (HAART) [2-6], leading to additional HIV-related morbidity and mortality. IRIS is characterized by acute inflammatory responses to chronic opportunistic infections such as schistosomias, and is accompanied by a state of immunosuppression, with the immune system beginning to recover with a response to a previously acquired opportunistic infection which results in a heightened inflammatory response that ironically makes the symptoms of infection worse [2,3,7-12]. The condition manifest with a wide range of clinical presentations and is associated with a number of antigenic targets [13,14], including, for example, antigens from viable replicating infective pathogens during a sub-clinical infection (unmasking IRIS), or from dead pathogen debris and dying of non-infective pathogens (paradoxical IRIS), host antigen (autoimmune disease), tumor antigen and other inflammatory conditions [13-15]. The pathogenesis of IRIS is poorly understood, however, monocytes and natural killer (NK) cells involved in innate immunity, inappropriate function of T-cell, polymorphism in human leukocyte antigen (HLA) and cytokine-related genes, have been associated with this clinical condition [16,17].

IL-23R is a type 1 trans-membrane protein found on dendritic cells, monocytes, activated T-cells and NK cells [18]. The IL-23R is a heterodimer, comprising the IL-12Rβ1 and a novel sub-unit named IL-23R [18]. The IL-12Rβ1 and the IL-12Rβ2 sub-units also form the IL-12 receptor (IL-12R). The IL-23R has an extracellular domain made of a signal sequence, an N-terminal immunoglobulin-like domain, and two cytokine receptor domains. The intracellular domain of IL-23R has seven tyrosine residues that can be phosphorylated. Three of the residues are *src* homology, two are domain-binding sites while two are signal transducers and activators of transcription (STAT) binding sites [19]. Binding of IL-23 to its receptor causes activation of Janus kinases (Jaks) which phosphorylates IL-23R at certain locations, thus forming docking sites for the STATs, and further enabling them to translocate to the nucleus where transcription of pro-inflammatory genes such as IL-17 and interferon-γ (IFN-γ) are initiated [19].

IL-23 is responsible for the differentiation and proliferation of Th_17_/Th_IL-17_ cells from naive CD4^+^ T cells [20]. Th_17_ aids in pathogen clearance and tissue inflammation by expressing elevated levels of the pro-inflammatory cytokine (IL-17) in response to stimulation, in addition to IL-1, IL-6, TNF-α, IL-22, and IL-25 (IL-17E) [21]. Other studies have also shown that IL-23-deficient mice were resistant to central nervous system’s (CNS) autoimmune inflammation because they were unable to develop IL-17 producing Th_IL-17_ cells [22]. Genome-Wide Association Studies (GWAS) have also established the IL-23R gene, as the susceptibility locus associated with some chronic inflammatory diseases, such as Crohn’s disease, inflammatory bowel disease and psoriasis [23-27], implicating this receptor in inflammatory diseases. However, to date, the functional associations between the IL-23 receptor (IL-23R) variants and susceptibility to Schistosoma-related IRIS in populations resident in Lake Victoria remains unknown. We hypothesized that the genetic variants within the synonymous (point mutations) and non-synonymous (an insertion or deletion of a single nucleotide in the sequence during transcription leading to a frameshift mutation) IL-23R gene would be predisposing factors for susceptibility to Schistosoma-related IRIS. Five polymorphisms were selected based on the frequency of mutant alleles (>10%) in the reference African Yoruba population following previous studies that have demonstrated that genes with mutant alleles having high frequencies are likely to be undergoing disease selective pressure [28,29]. The hallmark of HIV infection is characterized by depletion of CD4+ T cells and concomitant increase in HIV load [30,31]. IL-23 promotes proliferation of memory CD4+ T cells which are preferentially infected by HIV [19,32]. Despite years of intensive research, the mechanisms of CD4+ T cells depletion by the virus has remained widely speculative and it remain unclear whether or not, variation in the IL-23 or its receptor genes, could play a role in IRIS pathogenesis. Our current findings demonstrate that none of the IL-23R variants were associated with changes in CD4+ cells or HIV load during HAART. Results further demonstrated that carriage of the TT genotype at the rs1884444 T > G relative to GG, was associated with a decreased risk of schistosomiasis-associated IRIS.

## Methods

### Study population

The study targeted the fishing community in Uyoma, Rarieda District, along the shores of Lake Victoria in Kenya, a group occupationally-exposed to water infested with the infective stage of *Schistosoma mansoni* parasite. The prevalence of schistosomiasis in this population is high with about a third of them HIV-1 co-infected [33-36].

The following inclusion criteria were employed during recruitment of the study participants: participants had to be ≥18 years of age, be permanent resident of the study area, and willing to sign informed consent form. Other criteria for inclusion were: having undergone voluntary HIV counseling and testing in a recognized government institution, be HAART naïve at the beginning of the study, must have been screened for and had a history of treated schistosomiasis. Exclusion criteria included presence of other most common co-infections (e.g. malaria, tuberculosis, hepatitis B) in the populations that may independently dysregulate immune responses.

HAART naïve individuals were consented, recruited and underwent parasitological screening for schistosomiasis and Voluntary Testing and Counseling (VCT) for HIV prior to enrollment in HAART (this involved prescription of a combination of three drugs: lamivudine, stavudine and nevirapine) in a health care provider of their own choice.

### Ethical considerations

Prior to initiation of the study, the scientific and ethical clearance was obtained from the Scientific Steering Committee (SSC number 1763) and Ethical Review Committee (ERC), respectively, based at the Kenya Medical Research Institute (KEMRI).

### Laboratory testing and evaluations

Laboratory testing and evaluations were performed at baseline, after one month and three months post-enrollment. Schistosomiasis parasitological screen was performed by Kato Katz thick stool smear as previously defined [37]. However, data on HIV load and CD4 cell counts were both collected at baseline and three months post-HAART. The CD4 cell counts were performed using BD TruCount (BD Bioscience, San Jose, California) while HIV loads were determined using Amplicor HIV-1 Monitor Test (version 1.5) (Roche, Basel, Switzerland) from plasma stored at −80°C. Malaria parasitemia was determined by microscopy using Giemsa-stained blood smears while tuberculosis was determined using the standard tuberculosis skin test (PPD Skin Test). The Hepatatis B testing was conducted serologically using the Hepatatis B blood panel tests (Core Technology, Beijing, China), as per manufacturer’s instructions.

Schistosomiasis-associated IRIS was defined as either the re-emergence of symptoms of otherwise successfully treated schistosomiasis on initiation of HAART or the production of schistosome eggs by individuals who were not producing eggs before initiating HAART [38]. The chronic symptoms of schistosomiasis-associated IRIS included at least three of the following: hepato-splenomegaly; ultra-sound finding or clinical signs of hypertension; ultra-sound finding or clinical signs of increased granuloma formation/liver fibrosis; haematuria; and egg production in stool (whose presence could not be explained by other possible cause). Laboratory and clinical data were obtained at each visit and stored in electronic data base.

### Polymorphism genotyping

Genomic DNA was isolated from 2 mL of peripheral whole blood using Qi-Amp Midi kit (Qiagen, Hilden, Germany) as per manufactures instructions. A total of 5 polymorphic sites within the IL-23R were selected based on the mutant’s allele frequencies of over 10% in reference African Yoruba population (dbSNP: http://www.ncbi.nlm.nih.gov/SNP/, HAPMAP: http://www.hapmap.org/index.html.en). The IL-23R polymorphisms identified were rs7530511, rs1884444, rs11465754, rs6682925 and rs7539625. The polymorphic variants were determined using TaqMan 5’ Allelic Discrimination–Assay-By-Design (Applied Biosystems, Foster City, CA). Briefly, the assays were performed in a total volume of 5 μl (containing Taqman Universal Master Mix and SNP Genotyping Assay Mix) with the following amplification protocol: 95°C for 10 min, followed by 40 cycles of 95°C for 15 sec and 60°C for 1 min. Allele-calling was carried out using allele-specific fluorescence on the ABI StepOnePlus real-time PCR system (Applied Biosystems, Foster City, CA, USA). The automated sequence detection software (SDS) was then used for allelic discrimination (Applied Biosystems, Foster City, CA).

### Statistical analysis

Data was analyzed in SPSS (version 17.0 Chicago, IL). Proportions and allele frequencies between IRIS patients and controls were assessed through the use of Pearson χ^2^ and Fisher’s exact tests while Kruskal-Wallis test was used to analyze differences across groups. Medians were compared using the Mann–Whitney U test. Logistic regression analyses were used to calculate odds ratios (OR), 95% confidence interval (CI) and corresponding *P*-values for the association of each variant and susceptibility to IRIS, while controlling for the confounding effects of age and sex. These confounders were controlled for based on previous studies which have shown that they independently alter cytokine levels in disease [39]. The dependent variable in the regression analyses was the development of IRIS while the independent variables were the IL-23R genotypes. All *P* ≤ 0.05 were considered statistically significant.

## Results

### Demographic characteristics of the study participants

A total of ninety adults (n = 90) eligible patients were identified, 19 individuals were excluded from the study due to incomplete laboratory data. Of the remaining 71 study participants, 35 (49.3%) were males while 36 (50.7%) were females. A total of 26 (36.6%) of the patients developed IRIS while forty five (63.4%) did not develop IRIS and were used as controls. The distribution of the males *vs.* females (*P* = 0.154), median age (*P* = 0.867), baseline CD4 cell counts (*P* = 0.101) and baseline HIV plasma viral loads (*P* = 0.116) were comparable between the IRIS and non-IRIS patients. These findings demonstrate that demographic characteristics between the cases and the controls were fairly comparable in the study population (Table 1).

**Table 1:** Demographic characteristics of the study participants

	**IRIS**	**NON-IRIS**	***P-value***
Age at enrolment, years	35.5 (28–45)	34 (29–41)	0.867^a^
Male (n) (%)	14 (53.8)	21 (46.7)	0.154^b^
Base-line CD4 count	171.5 (112–251)	216 (144–317)	0.101^a^
Base-line Viral Load	93.2 (9.8-213)	234 (12.0-531)	0.116^a^
(10^3^xcopies/ml)			

Data are median and interquartile range unless stated otherwise. ^a^Statistical significance determined by Mann–Whitney U test. ^b^Statistical significance determined by Pearson’s Chi-square tests.

### Prevalence of IL-23R genotypes in *S. mansoni*-infected patients undergoing HAART

Samples from the 71 *S. mansoni*-infected individuals undergoing HAART were genotyped for the IL-23R polymorphisms (Table 2). Results revealed a comparable distribution of variants within the rs7530511 (*P* = 0.518), rs1884444 (*P* = 0.091), rs11465754 (*P* = 0.426), rs6682925 (*P* = 0.393), and rs6682925 (*P* = 0.088) between the IRIS and non-IRIS patients (Table 2).

**Table 2:** The prevalence of IL-23R genotypes in patients undergoing HAART (IRIS) and non-IRIS

**Genotype**	**IRIS**	**Non-IRIS**	***P*-*value***
**rs7530511**			
CC	17 (70.83)	33 (76.74)	0.518^a^
CT	5 (20.83)	9 (20.93)	
TT	2 (8.33)	1 (2.33)	
**rs1884444**			
GG	4 (18.18)	16 (39.02)	0.091^a^
GT	16 (72.73)	18 (43.90)	
TT	2 (9.09)	7 (17.07)	
**rs11465754**			
AA	7 (26.92)	16 (38.10)	0.426^a^
AG	16 (61.54)	19 (45.24)	
GG	3 (11.54)	7 (16.67)	
**rs6682925**			
CC	10 (38.46)	19 (48.72)	0.393^a^
CG	13 (50.00)	13 (33.33)	
GG	3 (11.54)	7 (17.95)	
**rs7539625**			
GG	5 (20.83)	16 (36.36)	0.088^a^
GA	17 (70.83)	19 (43.18)	
AA	2 (8.33)	9 (20.45)	

^a^Data are proportions (%) as determined by Fishers’ exact test. The targeted variants were those that had a prevalence of >10% in reference African Yoruba population (dbSNP and HAP-MAP). There were no significant differences in proportions of the variants in IRIS versus non-IRIS patients.

### Association between IL-23R genotypes and susceptibility to *S. mansoni*-related IRIS

In order to determine the association between IL-23R genotypes and susceptibility to *S. mansoni*-related IRIS, a multivariate logistic regression analyses was carried out while controlling for the confounding effect of age and sex. Results revealed that relative to the GG genotype, carriage of the TT genotype at the rs1884444 locus was associated with a reduced risk to IRIS (OR: 0.25, 95% CI: 0.07-0.96, *P* = 0.043). However, none of the other variants were associated with susceptibility to IRIS (Table 3).

**Table 3:** The association between IL-23R variants and susceptibility to IRIS

**IRIS**			
**Genotypes**	**OR**	**95% CI**	***P-value***
**rs7530511**			
CC	1.00		
CT	1.05	0.30–3.68	0.308
TT	3.64	0.30–43.62	0.386
**rs1884444**			
GG	1.00		
GT	0.24	0.04–1.51	0.129
TT	0.25	0.07–0.96	**0.043**
**rs11465754**			
AA	1.00		
AG	0.40	0.12–1.33	0.136
GG	0.37	0.07–1.84	0.222
**rs6682925**			
CC	1.00		
GG	1.95	0.64–5.94	0.240
GG	0.77	0.16–3.81	0.748
**rs7539625**			
GG	1.00		
GA	0.34	0.10–1.14	0.080
AA	0.25	0.05–1.40	0.116

Data are presented as Odds Ratio (OR) and 95% Confidence Interval (95% CI). Data analyzed by multivariate logistic regression analyses controlling for the confounding effects of age and sex.

*P*-values in bold are significant at *P* ≤ 0.05. The reference group in each of the analysis was the most prevalent genotype.

### The functional role of rs7530511, rs1884444, rs11465754, rs6682925, rs7539625 and changes in CD4 cell counts and HIV loads in blood

In order to determine the functional role of IL-23R variants in conditioning CD4 cell counts and HIV loads, the medians in cases versus controls were compared using Mann–Whitney U test, while across genotype comparisons was determined using Kruskal-Wallis test. Results in all the IL-23R variants demonstrated a marked decrease in HIV load coupled with marked improvement in CD4 cell counts. Additional across group comparison in genotypes revealed comparable levels in CD4 cell counts and HIV viral loads for variants in rs7530511 (*P* = 0.500, *P* = 0.405), rs1884444 (*P* = 0.414, *P* = 0.648), rs11465754 (*P* = 0.782, *P* = 0.975), rs6682925 (*P* = 0.700, *P* = 0.818), and rs7539625 (*P* = 0.715, *P* = 0.863), respectively (data not shown). Taken together, these results demonstrate that the presence of the IL-23R genotypes in cases or controls does not alter CD4 cell counts and HIV loads in this population.

## Discussion

This study investigated the association between five IL-23R variants and risk to schistosomiasis-associated IRIS in a Kenyan population naturally exposed to schistosomiasis. We demonstrate that the TT genotype of the rs1884444 (T > G) SNP in IL-23R gene was associated with a decreased risk of schistosomiasis-associated IRIS while the four other variant had no association with this disease condition in this western Kenyan population. The rs1884444 is located on exon 2 of IL-23R, and it has been previously shown to be responsible for the signal peptide of IL-23R, since variation in this site may interfere with binding of an exonic splicing enhancer leading to exon skipping, malformation or alternative splicing [40]. In addition, this polymorphism results in amino acid change in codon 3 (His > Gln) and may influence the ligand-receptor specificity and affinity [40], thereby modulating the pro-inflammatory effects of Th17 cell, thus leading to decreased risk to schistosomiasis-associated IRIS. It would be important to carry out a genome-wide study in IRIS-diseased versus non-diseased to fully identify potential genes that may modulate susceptibility to IRIS in populations naturally exposed to schistosomiasis.

Previous studies [41,42] demonstrated that IL-23/IL-17 pathway is essential for the development of severe schistosome egg-induced immunopathology and its absence cannot be compensated with other mechanisms. However, the exact molecular mechanism by which IL-23 regulates Th17 cell and the associated activities of the IL-23, IL-23R, and their SNPs and susceptibility to schistosomiasis-associated IRIS are undefined and require further studies. In the current study, we only managed to collect data from a total of 26 well-defined IRIS cases against 45 controls after three months post-HAART follow-up. This presented us with a challenge of harnessing enough numbers to effectively carry out detailed functional associations between the SNPs and differential changes in CD4+ cell counts and HIV viral load. Future studies should address the components of large numbers in a well-designed extensive longitudinal survey to fully elucidate the sample size effects over time.

Although previous studies had shown the importance of IL-23 in mediating the development of pathogenic CD4+ T-cell population that produces differential IL-17 (a cytokine associated with higher pathology in schistosomiasis), we found no association between the risk of schistosomiasis-associated IRIS and magnitude of change in CD4+ cell counts or decrease in HIV viral load. Even though surprising, this may be an indication that the rate of immune reconstitution is not an important factor in susceptibility to IRIS [19,43]. These findings contrast those of earlier studies, which demonstrated that marked reduction in viral load and magnitude of change in CD4+ cell counts is associated with IRIS risk [44]. The disparity in the current versus previous findings may be partly due to the relative frequency of various IRIS events across the two studies. In the previous studies, IRIS diagnosis was based on consensus expert opinion, and classified by mode of presentation (paradoxical worsening of known opportunistic infection [OI] or unmasking of sub-clinical disease) while in the current study, IRIS was defined as either the re-emergence of symptoms of otherwise successfully treated chronic schistosomiasis on initiation of HAART or the production of schistosome eggs by individuals who were not producing eggs before initiating them on HAART [38]. The symptoms of schistosomiasis-associated IRIS included at least three of the following: hepato-splenomegaly; ultra-sound finding or clinical signs of hypertension; ultra-sound finding or clinical signs of increased granuloma formation/liver fibrosis; haematuria; and egg production in stool (whose presence could not be explained by other possible cause). Such variations in regional definition of disease may lead to differences in both gene and functional associations. It may be plausible to develop regional definition to ascertain whether or not differences exist in disease manifestations.

## Conclusion

In conclusion, we demonstrate for the first time, in this case–control study from a high-risk Kenyan population that the non-synonymous SNP rs1884444 (His3Gln) of IL-23R gene was associated with a decreased risk of schistosomiasis-associated IRIS. However, none of the SNPs were functionally associated with CD4+ cells and HIV viral loads. Further studies incorporating functional evaluations of the IL-23/IL-23R on IL-23R carrier cells in larger cohorts are warranted to validate our findings.

### Ethical approval

The study was approved by the Ethics Review Committee of the Kenya Medical Research Institute. Informed written consent was obtained from all the participants in the study.

## Competing interest

The authors declare that they have no competing interests.

## Authors’ contributions

GOO, CO, ER, WGZOJ, EOM, DK, RC and PNM designed, carried out the survey studies in the population and participated in the drafting of the manuscript. GOO and CO performed the statistical analyses. All authors read and approved the final manuscript.

## Pre-publication history

The pre-publication history for this paper can be accessed here:

http://www.biomedcentral.com/1471-2334/14/316/prepub
